# The *MEROPS* database of proteolytic enzymes, their substrates and inhibitors in 2017 and a comparison with peptidases in the PANTHER database

**DOI:** 10.1093/nar/gkx1134

**Published:** 2017-11-14

**Authors:** Neil D Rawlings, Alan J Barrett, Paul D Thomas, Xiaosong Huang, Alex Bateman, Robert D Finn

**Affiliations:** EMBL European Bioinformatics Institute, Wellcome Trust Genome Campus, Hinxton, Cambridgeshire CB10 1SD, UK; Division of Bioinformatics, Department of Preventive Medicine, University of Southern California, 1450 Biggy St, NRT 2502, Los Angeles, CA 90033, USA

## Abstract

The *MEROPS* database (http://www.ebi.ac.uk/merops/) is an integrated source of information about peptidases, their substrates and inhibitors. The hierarchical classification is: protein-species, family, clan, with an identifier at each level. The *MEROPS* website moved to the EMBL-EBI in 2017, requiring refactoring of the code-base and services provided. The interface to sequence searching has changed and the *MEROPS* protein sequence libraries can be searched at the EMBL-EBI with HMMER, FastA and BLASTP. Cross-references have been established between *MEROPS* and the PANTHER database at both the family and protein-species level, which will help to improve curation and coverage between the resources. Because of the increasing size of the *MEROPS* sequence collection, in future only sequences of characterized proteins, and from completely sequenced genomes of organisms of evolutionary, medical or commercial significance will be added. As an example, peptidase homologues in four proteomes from the Asgard superphylum of Archaea have been identified and compared to other archaean, bacterial and eukaryote proteomes. This has given insights into the origins and evolution of peptidase families, including an expansion in the number of proteasome components in Asgard archaeotes and as organisms increase in complexity. Novel structures for proteasome complexes in archaea are postulated.

## INTRODUCTION

The *MEROPS* database, which is a manually curated information resource for proteolytic enzymes, their inhibitors and substrates, relocated to the EMBL-European Bioinformatics Institute (EMBL-EBI) during 2017. The new URL for the website is http://www.ebi.ac.uk/merops/.

The hierarchical classification in *MEROPS* was established for peptidases in 1993 (1) and for peptidase inhibitors in 2004 (2). The classification involves the clustering of homologous sets of peptidase and protein inhibitor sequences into peptidase and inhibitor ‘species’ (represented by a unique identifier), which are in turn clustered into families, which are clustered into clans. A family contains related sequences, and a clan contains related tertiary structures. Sequence analysis is restricted to that portion of the protein directly responsible for peptidase or inhibitor activity, which is termed the ‘peptidase unit’ or the ‘inhibitor unit’. The peptidase unit includes primary substrate binding sites (though not necessarily secondary binding sites, known also as ‘exosites’) and the catalytic residues. The inhibitor unit is a domain that interacts with a peptidase domain and, if one exists, will include the residues that provide the reactive bond that occupies the active site. A peptidase or inhibitor unit normally corresponds to a structural domain, and some proteins contain more than one peptidase or inhibitor domain. Examples are the potato virus Y polyprotein, which contains three peptidase units, each in a different family and chicken ovoinhibitor, which contains seven inhibitor units all in the same family. At every level in the database a well-characterized type example is nominated, to which all other members of the family or clan must be shown to be related in a statistically significant manner. The type example at the peptidase or inhibitor level is termed the ‘holotype’ (1–2). Criteria for distinguishing one peptidase species from another were established in 2007 (3). For simplicity, the term ‘peptidase’ also includes isopeptidases and self-processing proteins such as asparagine lyases (4).

Each clan, family, holotype peptidase and holotype inhibitor is assigned to an identifier. For a clan, the identifier consists of two letters. The first indicates the catalytic type (‘A’ for aspartic peptidase, ‘C’ for cysteine peptidase, ‘G’ for glutamic peptidase, ‘I’ for inhibitors that are proteins, ‘M’ for metallopeptidase, ‘P’ for peptidases of mixed catalytic type, ‘S’ for serine peptidase, ‘T’ for threonine peptidase, ‘N’ for asparagine lyase, and ‘U’ for peptidases of unknown catalytic type. The second letter is assigned sequentially as each clan is identified. An example of a clan identifier is CA, which includes cysteine peptidases with a papain-like fold. For a family, the identifier consists of an initial letter, again corresponding to catalytic type, and a number. An example is C1, the family of papain-like cysteine peptidases. For a holotype, the identifier consists of the family name (padded with a zero when necessary to make it three characters long), a dot, and a number. An example is cathepsin B: C01.060. An identifier where ‘9’ follows the dot is a non-peptidase homologue (e.g. testin, C01.972). An identifier where ‘P’ follows the dot is a pseudogene (e.g. the cathepsin L-like pseudogene 1, C01.P02).

Among the criteria for distinguishing one peptidase from another is the action on substrates. A collection of known cleavage sites in substrates, including proteins, peptides and synthetic substrates, has been established (5). Similarly, a collection of peptidase-inhibitor interactions has also been established, which provides evidence for distinguishing peptidases and inhibitors (6). Because the MEROPS classification of inhibitors can only be applied to inhibitors that are proteins, a second, unclassified, collection of small molecule inhibitors was established (6).

In addition, the *MEROPS* database and website includes an extensive, manually curated bibliography. References are assigned to the relevant *MEROPS* identifiers so that there are references for each clan, family, peptidase, inhibitor, substrate cleavage and peptidase-inhibitor interaction.

The underlying principle behind the *MEROPS* database has been first to identify the activity corresponding to a proteolytic enzyme or a peptidase inhibitor in the scientific literature, and to assess the reliability of that claim. The criteria for acceptance of a claim of novel peptidase activity will be any of the following: that proteolytic activity is directly shown on a substrate; that the activity is either significantly different from any known activity or that although the activity is similar to a known peptidase the sequences are <50% identical. For an inhibitor, the alleged inhibitory activity must not be the consequence of a competing substrate. Once an activity is established as being novel, then that activity will become the holotype for a new *MEROPS* identifier. If the activity can be associated with a protein sequence, then sequence clustering is attempted. If the sequence can be shown to be similar to those in an established family, then the new *MEROPS* identifier will be an addition to that family. If the new sequence shows no significant relationship to any sequence in the *MEROPS* collection, then a new family is established. It is essential that the extent of the peptidase or inhibitor unit should be established. The new sequence is used to search the Pfam database (7) for relationships to any known domains, and these are excluded from the peptidase/inhibitor unit. We also exclude known or predicted signal and transit peptides, and known propeptides. Once the peptidase/inhibitor unit has been defined, it becomes the new family type example and it is used to search either the UniProtKB (8) or NCBI Protein sequence databases (9) with BLASTP (10) or via the HMMER 3 webserver (11). Any homologues found with a matching E-value of 0.001 or less, are assembled into the new family. Initially, these are given a temporary, miscellaneous *MEROPS* identifier, which is the family name followed by a dot and either ‘UPW’ for a homologue that has retained all the active site residues or ‘UNW’ for a homologue that has any active site residue replaced or missing. A sequence library is made from the peptidase/inhibitor units of the homologues found. An alignment is made from the sequence library using MUSCLE (12) and a phylogenetic tree is made from the alignment using the UPGMA method of QuickTree (13). From examining the phylogenetic tree, sequences that cluster around a holotype sequence are assigned the same *MEROPS* identifier as that of the holotype. A sequence that does not cluster with a holotype retains its miscellaneous identifier.

If the new activity cannot yet be associated with a protein sequence, then a special *MEROPS* identifier is assigned in which the first character indicates the catalytic type, the second character is ‘9’, and the third character is a letter, depending on the type of proteolytic activity (A indicates an aminopeptidase, B is a dipeptidase, C is a dipeptidyl-dipeptidase, D is a peptidyl-dipeptidase, E is a carboxypeptidase, F is an omega peptidase, and G is an endopeptidase). An example of such an identifier is M9A.007 (Xaa-Trp aminopeptidase, also known as aminopeptidase W) (14). Obviously, the activity cannot be added to an existing family and new families cannot be assembled without a protein sequence, but action on substrates and interaction with inhibitors can be added to our data, which may help with identifying the source sequence in the future.

A full methodology for how families and clans are assembled was published in 2014 (15). Statistics from release 11.0 (January 2017) of *MEROPS* are shown in Table 1 and compared with release 9.13 (July 2015). Counts of substrate cleavages, peptidase–inhibitor interactions and references are shown in Table 2.

**Table 1.** Counts of protein-species, families and clans for proteolytic enzymes and protein inhibitors in the *MEROPS* database

	MEROPS 12.0 (September 2017)		MEROPS 9.13 (July 2015)	
	Peptidases (change)	Inhibitors (change)	Peptidases	Inhibitors
Sequences	908 326 (384 4550	134 011 (59 353)	523 871	74 658
Identifiers (Total)	5267 (645)	868 (156)	4622	712
*experimentally characterized and sequenced*	3181 (387)	725 (128)	2794	597
*hypothetical from model organisms*	1407 (–221)	0	1628	0
*not active as peptidase or inhibitor*	357 (24)	115 (0)	333	115
*experimentally characterized but unsequenced*	215 (12)	0	203	0
*pseudogenes*	72 (2)	0	70	0
*Compound and complex proteins*	17 (1)	53 (-3)	16	56
Families	268 (15)	82 (3)	253	79
Clans	62 (1)	39 (0)	61	39

The numbers in Release 12.0 of *MEROPS* (September 2017) are compared to those in Release 9.13 of *MEROPS* (July 2015). A peptidase is referred to as ‘unsequenced’ when no sequence is known, or the known sequence fragments are insufficient to be able to assign the peptidase to a family. The number of identifiers for hypothetical peptidases from model organisms has decreased because many have now been experimentally characterized.

**Table 2.** Information in the MEROPS database

	MEROPS 12.0 (change)	MEROPS 9.13
Substrate cleavages: total	92 216 (27 746)	64 470
Substrate cleavages: physiological	22 100 (4969)	17 131
Substrate cleavages: non-physiological	59 485 (22 504)	36 981
Substrate cleavages: pathological	1413 (–13)	1426
Substrate cleavages: synthetic substrates	6016 (51)	5965
Peptidase-inhibitor interactions: total	6455 (253)	6202
Peptidase-inhibitor interactions: proteins	1486	1428
Peptidase-inhibitor interactions: SMI	4970	4419
References	64 647	59 155

Substrate cleavage totals do not include cleavages derived only from the SwissProt database (mainly removal of initiating methionines and signal peptides). A naturally occurring cleavage is described as ‘physiological’ when the peptidase and substrate are from the same organism, and ‘pathological’ if the organisms differ and are pathogen and host.

### New feature: cross-references to the PANTHER database

#### Methodology

The majority of databases that classify sequences do so on the basis of structural or sequence similarities, and sequences are classified into families. However, some proteins have diverged significantly in function even though structural or sequence similarities are still detectable, with the result that a family can include proteins with different functions. As described above, we attempt to provide a finer grade of classification by grouping sequences within a family into ‘protein-species’ each of which is given a unique *MEROPS* identifier. Previously, it had not been possible to establish mapping between *MEROPS* and another database at a level lower than family.

One database that attempts to provide is similar fine grain of classification is PANTHER (16). PANTHER also attempts to classify by sequence similarity (at the family level) but also to identify proteins within a family that represent an orthologous group and generally have a very similar function (at the subfamily level). The Panther subfamily is different to a *MEROPS* subfamily, which represents a cluster within a family that result from an ancient divergence (calculated from a phylogenetic tree to have occurred ∼2.5 billion years ago). A subfamily in *MEROPS* is usually the result of the merging of what had been separate families.

The principles used at PANTHER are to assemble a family of sequences with similarity identified by HMMER searches, and to generate an alignment using MAFFT (17) and from it to construct a phylogenetic tree using GIGA (18). GIGA reconciles the gene tree to a guide species tree, identifying nodes in the tree as representing gene duplication, horizontal transfer and speciation events. For each gene duplication or horizontal transfer, a new subfamily is established. By examining the tree, gene duplications that precede speciation events can be discovered. For each gene duplication, a new subfamily is established. As described above, a similar procedure takes place at *MEROPS*. Cross-references between MEROPS and PANTHER can therefore be identified by examining which sequences are common to clusters from each database, or more accurately by identifying in which PANTHER subfamily a *MEROPS* holotype sequence occurs. Both approaches have been attempted. There is mutual advantage in identifying circumstances when these do not concur, because it helps curators of both databases to discover false positives, and to refine their classification systems.

There are several major differences between *MEROPS* and PANTHER methodologies that hamper a direct comparison. (i) PANTHER includes all proteins, whereas MEROPS is restricted to peptidases and peptidase inhibitors. (ii) *MEROPS* includes sequences from all organisms, whereas the PANTHER analysis is restricted to 103 ‘reference’ organisms. (iii) *MEROPS* analyses are based on the peptidase or inhibitor unit only, whereas PANTHER analyses are based on full-length sequences. Because many peptidases and inhibitors are multidomain proteins, and the domains and their arrangement are not necessarily the same for members of a single family, we expect a one-to-many relationship between *MEROPS* and PANTHER families. On the other hand, a single *MEROPS* identifier should correspond to a single PANTHER subfamily.

#### Cross-references at the family level

A total of 332 PANTHER families can be mapped to 203 *MEROPS* families and subfamilies (see Table 3), because of the many-to-one relationship between PANTHER and *MEROPS* families. There are 141 *MEROPS* families and subfamilies that map to a single PANTHER family, and 62 *MEROPS* families and subfamilies that map to more than one PANTHER family. The more sequences assigned to a *MEROPS* family, the more likely it is that the *MEROPS* family will represent more than one PANTHER family. The largest *MEROPS* subfamily is S1A (the chymotrypsin subfamily containing 42 715 sequences) which is mapped to 40 PANTHER families. Other large *MEROPS* families and subfamilies that map to many PANTHER families are: S33 (28,613 sequences) mapped to five PANTHER families; S8A (subtilisin; 26 501 sequences) mapped to six PANTHER families; and C19 (deubiquitinating enzymes; 25,788 sequences) mapped to 37 PANTHER families. The *MEROPS* subfamily containing most sequences (15 234) that is mapped to a single PANTHER family is T1A (proteasome) mapped to PTHR11599. The *MEROPS* families with fewest sequences that is mapped to more than one PANTHER family is N9 (an asparagine lyase; 90 sequences), mapped to PTHR15184 and PTHR43607. *MEROPS* identifiers for uncharacterized peptidase homologues from model organisms, and non-peptidase and non-inhibitor homologues are mapped to PANTHER subfamilies, but not to the PANTHER family unless other subfamilies map to characterized peptidases and inhibitors. Similarly, any cross-reference to only one of several subfamilies in a PANTHER family were manually checked, and not mapped at the family level unless all subfamilies were annotated as peptidases or inhibitors in PANTHER.

**Table 3.** Comparison of the *MEROPS* and PANTHER databases

	*MEROPS*	PANTHER	Overlap	%	Unique to *MEROPS*	%
Families	428	14710	203	47.43	225	52.57
Identifiers/subfamilies	4989	76032	2920	58.56	2069	41.47

Counts of the different entry types from the databases are shown. Percentages are calculated only for the MEROPS database.

Because PANTHER aligns full sequences, whereas *MEROPS* aligns only sequences of the peptidase or inhibitor domains, a cluster of homologous sequences each of which contains more than one peptidase or inhibitor domain can be represented by a single PANTHER family and more than one *MEROPS* family. Examples are: S8 and I9 (the subtilisin family and the inhibitory subtilisin propeptide), both of which correspond to PTHR10795; C1 and I29 (the papain family and its inhibitory propeptide) which equate to PTHR12411; and sequences from I1 and I31 are found in PTHR44341. Because *MEROPS* families S55 is closely related to S1C and S1D, all are represented by PTHR22939. Some multidomain peptidases share domains other than peptidase domains despite being from different *MEROPS* families, and these sequences can also map to a single PANTHER family. Examples include sequences from M43B and S1A that share a sushi domain and are included in PTHR19325; and M15D and S12 in PTHR22935 (penicillin-binding domain). Sequences from C80, S9B and S9C that share a domain of unknown function are included in PTHR12277, as do sequences from C39 and S8A in PTHR24221.

Of the cross-references that have been established at the *MEROPS* subfamily level, there is only one instance where all the *MEROPS* subfamilies in a family are combined in a single PANTHER family: A1A (pepsin) and A1B (nepenthesin) in PTHR13683.

#### Unmatched *MEROPS* families

There are 224 *MEROPS* families and subfamilies that have no PANTHER equivalent. Some of these will not appear in PANTHER, because: (i) the family has not been built yet; (ii) they are exclusively from viruses and PANTHER does not include any viruses in its set of reference organisms (80 *MEROPS* families and subfamilies); (iii) PANTHER requires five sequences to build a family and there are 34 *MEROPS* families and subfamilies that contain less than five sequences (of which 18 are peptidase inhibitors and 13 are exclusively from viruses). Thus, 123 MEROPS families and subfamilies could be included in PANTHER. The largest of these with 13 578 sequences is C40 (dipeptidyl-peptidase 6), which is predominantly bacterial but with some eukaryote sequences. This family includes 447 sequences from model organisms in the PANTHER set. Families such as C40 have not yet been built by the PANTHER team, and we are currently exchanging the data necessary to add them. This emphasizes the advantages of the collaboration between a generalist database such as PANTHER and a specialist database such as *MEROPS*.

#### Cross-references at the subfamily level

It has been possible to establish cross-links between a *MEROPS* identifier and a PANTHER subfamily for 2,925 identifiers out of the 4924 for which holotype sequences have been defined, or 59.4%. There are 1744 *MEROPS* identifiers representing characterized peptidases and inhibitors that do not match a PANTHER subfamily. Of these, 188 are from viruses, and a further 1196 are from organisms not in the PANTHER set. There are 897 *MEROPS* identifiers which are each assigned to less than five sequences, and are below the threshold for creating a subfamily in PANTHER. The species with most *MEROPS* holotypes not in PANTHER are shown in Table 4. For plants such as barley and potato, parasites such as *Ancylostoma caninum*, and the crayfish, most of the *MEROPS* holotypes missing from PANTHER are peptidase inhibitors; for snakes such as *Bothrops jararaca* and *Gloydius halys* the missing holotypes are mostly peptidases from their venoms. At the time of writing, no venomous snake has had its genome completely sequenced, and parasite genomes and proteomes are frequently omitted from analyses because they are assumed to be degenerate. Although there is convenience, particularly in reducing computer time and overheads for analysis, in using restricted sets of organisms, there is an inherent danger that important biological aspects will be overlooked, so knowing the sources from which most proteins have been characterized will help with expanding the set of reference organisms in PANTHER.

**Table 4.** Commonest source organisms for *MEROPS* holotypes not in the PANTHER database

Holotypes	Species (common name)	MEROPS identifiers
23	*Hordeum vulgare* (barley)	A01.020, C01.024, C01.041, C01.168, I03.004, I04.032, I04.065, I06.002, I12.009, I12.010, I12.950, I12.951, I13.003, I13.005, I25.051, I25.052, I25.053, I25.054, I25.055, I25.056, I25.057, I25.059, I25.060
21	*Toxoplasma gondii*	A01.087, A01.098, C01.071, C01.149, C01.165, I01.024, I01.025, I01.026, I01.027, I01.041, I81.001, I82.001, M16.021, M16.022, S08.141, S08.154, S54.019, S54.020, S54.021, S54.022, S54.023
15	*Solanum tuberosum* (potato)	I03.002, I03.020, I13.002, I13.006, I20.001, I20.950, I25.015, I25.034, I25.035, I25.036, I25.037, I25.038, I25.039, I25.040, I37.001
14	*Pacifastacus leniusculus* (signal crayfish)	I01.039, I01.963, I01.964, I01.965, I19.002, I19.003, I19.004, I19.005, I19.006, I19.007, I19.008, I19.009, I19.010, S01.413
14	*Porphyromonas gingivalis*	C02.022, C10.002, C10.003, C25.001, C25.002, C25.003, C25.004, M13.009, M14.023, S01.527, S09.017, S09.075, S46.001, U32.001
13	*Bothrops jararaca* (Jacaraca snake)	I25.026, M12.138, M12.139, M12.140, M12.170, M12.304, M12.305, M12.320, S01.179, S01.180, S01.353, S01.354, S01.433
12	*Ancylostoma caninum*	I02.034, I02.957, I02.958, I02.960, I02.961, I02.962, I02.963, I02.964, I02.965, I08.010, M12.310, M13.011
12	*Aspergillus fumigatus*	A01.026, A01.077, I78.001, M03.009, M19.013, M28.022, M36.001, M77.001, M77.002, M77.003, M77.004, S09.012
12	*Gloydius halys* (Halys pit viper)	M12.022, M12.134, M12.178, M12.315, M12.326, S01.253, S01.331, S01.332, S01.333, S01.338, S01.350, S01.497
12	*Xenopus laevis* (African clawed frog)	M12.014, M12.015, M12.213, M12.238, S01.022, S01.024, S01.048, S01.049, S01.050, S01.126, S01.240, S01.245
11	*Lactococcus lactis*	C01.086, C39.007, C40.006, M01.002, M03.007, M13.004, S08.019, S08.059, S08.116, S15.001, U27.001
11	*Manduca sexta* (tobacco hawkmoth)	I04.031, I04.066, I04.083, M01.013, M01.030, S01.018, S01.040, S01.427, S01.444, S01.467, S01.484
10	*Streptococcus pyogenes*	C10.001, C39.003, C51.002, C60.003, C60.006, C66.001, I69.001, I69.002, S08.020, S08.027
10	*Streptomyces lividans*	M01.009, M07.001, S08.091, S12.001, S26.025, S33.002, S33.006, S33.007, S33.992, S37.001

The number of holotypes, source organism and MEROPS identifiers are shown.

A PANTHER subfamily can represent more than one *MEROPS* identifier. For example, if an inhibitory protein contains more than one inhibitor unit, there may be a *MEROPS* identifier for each unit, but only one PANTHER subfamily accession for the whole protein. For example, PANTHER subfamily PTHR10913:SF45 represents ovoinhibitor, which has seven inhibitor units assigned the *MEROPS* accessions I01.004 to I01.010. However, there are instances where a PANTHER subfamily accession corresponds to more than one MEROPS identifier, even though there is only one peptidase unit in each of the proteins. This can occur when a gene duplication has preceded speciation, but different characteristics have subsequently evolved. For example, PTHR11533:SF205 corresponds to *MEROPS* identifiers M01.006 (Ape2 aminopeptidase from fungal mitochondria), M01.007 (Aap1΄ aminopeptidase from fungal cytoplasm), M01.010 (cytosol alanyl aminopeptidase from animal cytoplasm) and M01.015 (aminopeptidase H11 from nematodes, which is a type II membrane protein): the proteins differ in their localization even though the genes are orthologous. If gene duplication occurs after speciation, but only in a limited set of organisms, there may not be enough examples in the PANTHER organism set to establish an identifier. For example, PTHR10201:SF151 corresponds to *MEROPS* identifiers M10.001 (matrix metallopeptidase-1; MMP1), M10.033 (rodent collagenase-like A peptidase) and M10.034 (rodent collagenase-like B peptidase); the gene duplication of *mmp1* is confined to rodents.

#### Identifying new MEROPS holotypes

Because PANTHER may divide a family into subfamilies, and not all subfamilies within a family map to a *MEROPS* holotype, a sequence from an unmapped PANTHER subfamily could theoretically represent a new *MEROPS* holotype for an uncharacterized protein. There are 1136 subfamilies from 134 PANTHER families mapped to *MEROPS* families that are not mapped to *MEROPS* identifiers. We are investigating the possibility of adding additional model organisms to the *MEROPS* set from the PANTHER set to enable us to create more holotypes.

**Figure 1.**
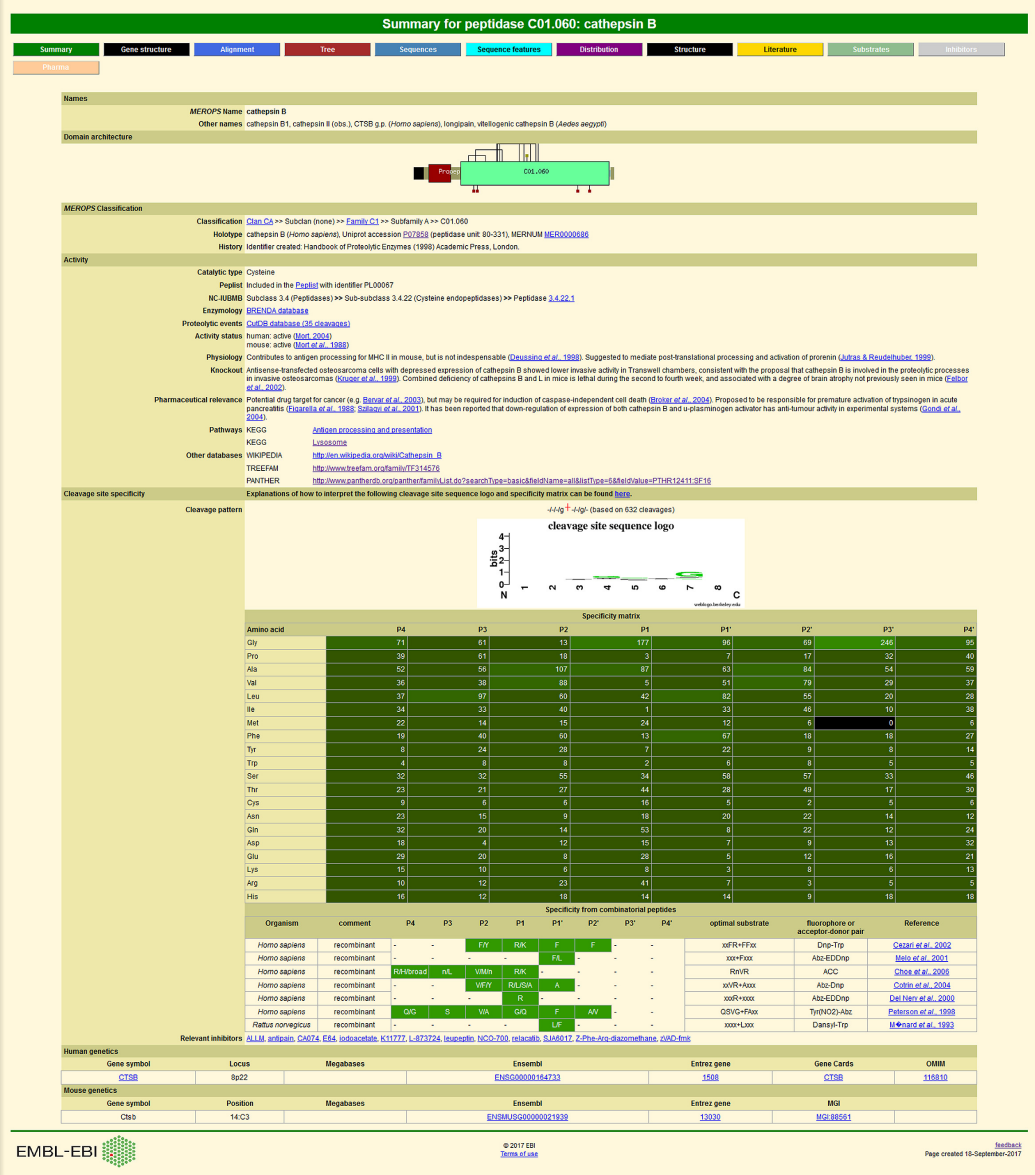
An example peptidase summary page showing cross-references to external databases. The page displaying the summary for cathepsin B is shown and includes cross-references to the Enzyme Nomenclature, TreeFam and PANTHER databases.

Cross-references from *MEROPS* to the PANTHER website are shown on the family, peptidase and inhibitor summary pages of *MEROPS* (see Figure 1). Reciprocal cross-references will also appear in the next release of PANTHER.

### Changes to existing features and methodologies

#### Sequence searching

The *MEROPS* sequence libraries have been made available via the protein ‘Sequence Similarity Search’ pages on the EMBL-EBI website (http://www.ebi.ac.uk/Tools/sss/). The *MEROPS* libraries can be found under the ‘Other Protein Databases’ tag. There are three *MEROPS* sequence libraries: MEROPS-MPRO, which contains full-length sequences for all the proteins in the *MEROPS* collection; MEROPS-MPEP, which contains only the sequences of the peptidase and inhibitor units from all the sequences in the *MEROPS* collection; and MEROPS-MP, which contains the sequences of peptidase and inhibitor units from all *MEROPS* family and subfamily type examples, and all holotypes. We recommend that a search is first performed against MEROPS-MP to identify that a protein sequence is a peptidase or inhibitor homologue, and then against either of the other two libraries to discover whether or not the sequence is in our collection. The *MEROPS* libraries can be searched with BLASTP (http://www.ebi.ac.uk/Tools/sss/ncbiblast/), PSIBLAST (http://www.ebi.ac.uk/Tools/sss/psiblast/) or FastA (http://www.ebi.ac.uk/Tools/sss/fasta/).

It is now also possible to search the *MEROPS* sequence library with HMMER using phmmer (https://www.ebi.ac.uk/Tools/hmmer/search/phmmer), hmmsearch (https://www.ebi.ac.uk/Tools/hmmer/search/hmmsearch) or jackhmmer (https://www.ebi.ac.uk/Tools/hmmer/search/jackhmmer) by selecting ‘MEROPS’ from ‘Current database selection:’. This repertoire of searches have been introduced because using a profile HMM search is typically more sensitive and faster than a BLAST search. If a search against UniProtKB returns peptidase or peptidase inhibitor sequences, the user is advised to search the *MEROPS* sequence library for more information and annotation. For a known peptidase or protein inhibitor sequence, the user can search UniProtKB with HMMER, quickly returning all known homologues.

Unfortunately, because a maximum sequence limit imposed by EMBL-EBI, the existing *MEROPS* batch Blast (19) has been suspended. We are working on a replacement service.

#### *MEROPS* sequence accessions

We are now using HMMER3 as well as BLASTP to assemble protein families. The most recent search of the UniProtKB database using *MEROPS* family type example sequences and HMMER3 returned >500 000 additional peptidase and inhibitor homologues. The number of sequences annotated in the *MEROPS* database has doubled since release 9.13, and this has forced a change in the accession number for each sequence. We have added an extra digit, so that the accession is now ‘MER’ followed by seven digits. For existing accessions we have added a zero after ‘MER’, for example, the human pepsin A sequence is now MER0000885.

#### Protein tertiary structures

A new method has also been implemented to retrieve tertiary structures from the PDB database (20). This has resulted in the addition of over 8500 new cross-references. The structure pages have also been modified. Links to obsolete resources have been replaced by links to PDBe (21) and PDBSum (22).

#### Adding sequences to the database

With almost a million sequences now included in MEROPS, adding more uncharacterized, hypothetical homologues would seem to present little reward for the effort involved. Many will be minor variants of existing sequences, and most will be derived from whole genome sequencing projects and unlikely to be characterized biochemically. As more sequences are added to existing alignments and trees, even lists of homologues, these became less useful to the user. We have taken the decision to only add sequences of characterized proteins and hypothetical sequences from organisms that are of evolutionary, medical or economic interest. Should a user want to identify all the known homologues of a peptidase or protein inhibitor, then we advise a search on the HMMER website against UniProtKB.

Examples of organisms of evolutionary interest include members of the recently described Asgard superphylum of Archaea, of which the Lokiarchaeota have been postulated as the closest relatives of the ancestral, pre-mitochondrial eukaryote (23,24). These organisms have never been isolated and the genomes have been assembled from metagenomics studies of ocean sediments (Lokiarchaeota, Heimdallarchaeota) and hot springs (Odinarchaeota). To see if the peptidases from Asgard archaeotes and eukaryotes are closely related, and to help understand the origin of peptidase families and folds, the proteomes of four Asgard archaeans have been searched for peptidase homologues. The results are compared to those from the well-known archaeans *Pyrococcus furiosus* (a euryarchaeote) and *Sulfolobus acidocaldarius* (a crenarchaeote) as well as the fission yeast *Schizosaccharomyces pombe* (chosen because it is a well-known single-celled eukaryote with a small genome size that is not a pathogen or symbiont and therefore unlikely to have a degenerate proteome) and the bacterium *Pelagibacter ubique* (chosen because it has been identified as closely related to the ‘proto-mitochondrion’ and is not pathogenic, unlike *Rickettsia*) (25). The results are shown in Supplementary Table S1. For all eight organisms, peptidase homologues are encoded by between 2.1% (Lokiarchaeote) and 4.0% (*P. furiosus*) of the genes in each genome. Eight families are present in all eight organisms, and were therefore probably present in the last common ancestor: M20 (glutamate carboxypeptidase), M24 (methionyl and X-Pro aminopeptidases), M38 (mostly non-peptidase homologues such as dihydro-orotase but also an isoaspartyl dipeptidase), C44 (the self-processing amidophosphoribosyltransferase and glutamate synthase precursors), T1 (proteasome), S8 (subtilisin, furin), S9 (prolyl oligopeptidase, dipeptidyl-peptidase IV, acylaminoacyl-peptidase), and S33 (prolyl aminopeptidase). Four families are present only in Asgard archaeotes and the fission yeast, and were therefore probably present in the proto-eukaryote: C14 (caspase), M3 (thimet oligopeptidase), M14 (carboxypeptidase A), and T5 (the self-processing ornithine acetyltransferase precursor). Three families, all of them metallopeptidases, are present in *P. ubique* and *S. pombe* and may therefore have been derived in eukaryotes from the proto-mitochondrion: M41 (FtsH peptidase), M16 (pitrilysin) and M17 (leucyl aminopeptidase). No families have homologues shared only by *S. pombe* and either *P. furiosus* or *S. acidocaldarius*, so this supports the idea that eukaryotes are more closely related to Asgard archaeota than either euryarchaeotes or crenarchaeotes.

There are, however, many important peptidase families and clans that are found in fission yeast and most eukaryotes but which are not found in the prokaryotes shown in Supplemtary Table S1. These include the pepsin family A1 and other families with the same fold (clan AA); families in clan CA, which includes papain-like cysteine peptidases and deubiquitinating enzymes; family M12 (astacins and reprolysins); and family S10 (serine carboxypeptidases). The absence of family C19 deubiquitinating enzymes from Lokiarchaeotes is particularly intriguing, because an otherwise complete ubiquitin-degradation pathway was identified (25). Bacterial homologues are known for some of these (shewasins from *Shewanella* in family A1; aminopeptidase C from *Lactobacillus* in clan CA; flavastacins from family M12; numerous uncharacterized homologues from family S10). Archaean homologues are known for several families in clans AA and CA. The presence of these families and clans in both prokaryotes and eukaryotes may be the result of horizontal gene transfer, though the direction of transfer may be unclear.

**Figure 2.**
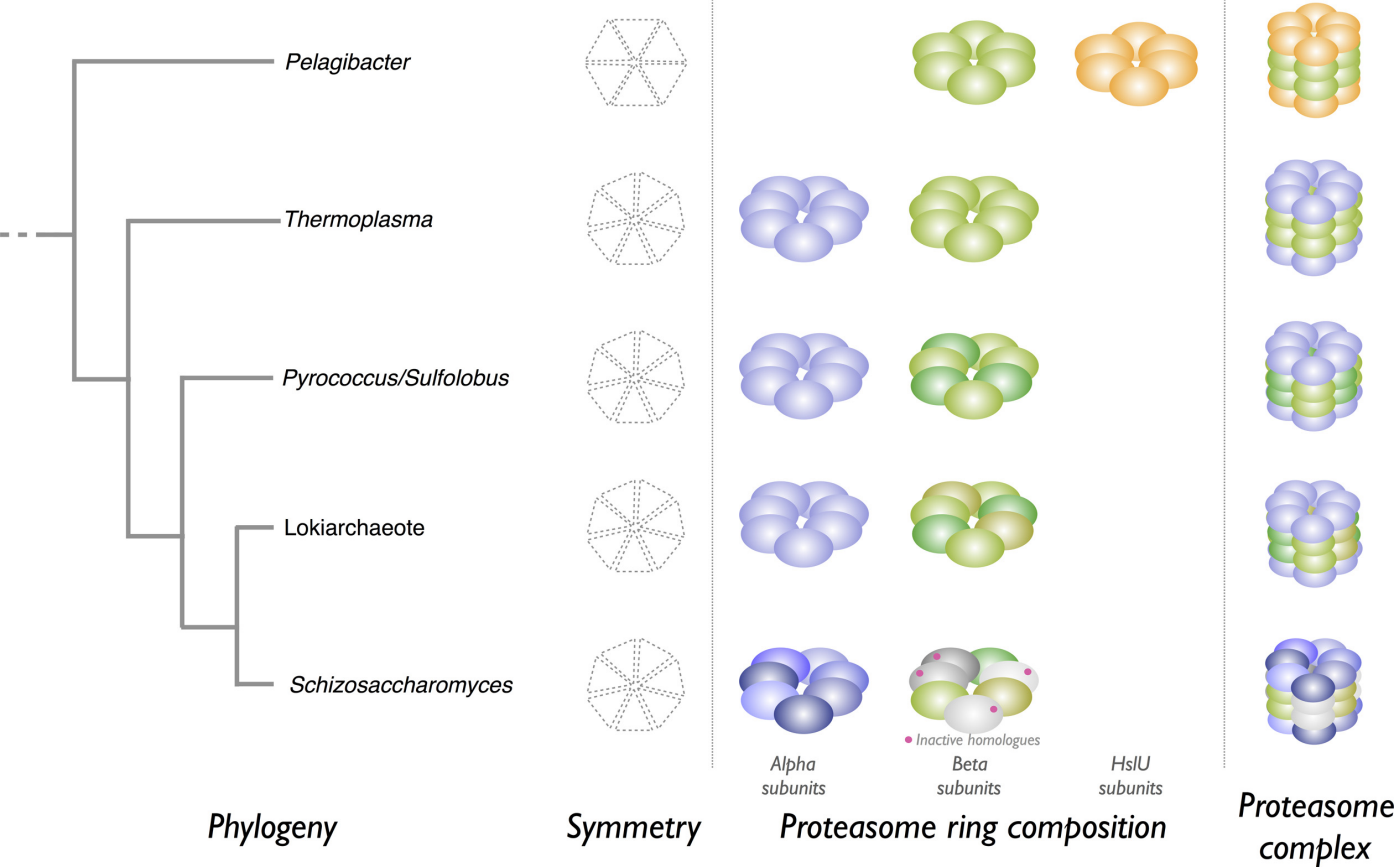
Examples of proteasome complexes. Schematic representations of proteasome complexes from selected organisms are shown. The predicted relationship between the organisms is shown as a simplified phylogenetic tree at the left of the figure. The symmetry of each ring, ring composition and structure of the complexes are shown; these are hypothetical for proteasomes from *Pyrococcus*, *Sulfolobus* and the Lokiarchaeote. Homologues of peptidase family T1 are shown as filled spheres: alpha subunits are shown in shades of blue, and beta subunits in shades of green (proteolytically active subunits) and grey (proteolytically inactive). The unrelated HslU subunits are shown as orange spheres.

Expansion of families in some species is also observed. One of the most intriguing expanded families is T1, the proteasome. In all species, the proteasome is a multisubunit complex forming a hollow cylinder, and substrate proteins are denatured and threaded through the proteasome into the central cavity where proteolysis takes place. In bacteria, such as *P. ubique*, the single proteasome homologue is a component of the HslUV complex. The other component is HslU, which is an ATPase and unrelated to peptidases. HslUV consists of four rings, each a homoheptamer, and stacked in the order HslU, HslV, HslV, HslU (26). In the archaean *Thermoplasma acidophilum*, the arrangement is similar except that rather than being a homohexamer, each ring is a homoheptamer of a single alpha or beta subunit, stacked in the order alpha, beta, beta, alpha (27). In *S. pombe* and all eukaryotes there are at least fourteen homologues. The eukaryote proteasome is a complex of 28 subunits, two of each homologue. Rather than being a homoheptamer, each ring is a heteroheptamer of seven alpha or seven beta subunits, stacked in the order alpha, beta, beta, alpha. Only three of the beta subunits are peptidases; none of other subunits have any proteolytic activity (28). Unlike *T. acidophilum*, the archaeans *P. furiosus* and *S. acidocaldarius* have a second beta subunit. Intriguingly, the number of subunits in the Asgard archaeotes is even greater, raising the possibility that proteasomes exist in these species that are more complex than in *T. acidophilum* but less complex than in eukaryotes. A schematic of these complexes is shown in Figure 2, but the exact arrangement of beta subunits in *P. furiosus, S. acidocaldarius* and Lokiarchaeotes is not known and that shown in the figure is speculation.

## Supplementary Material

Supplementary Data
