# Supplementary material for: The MEROPS database of proteolytic enzymes, their substrates and inhibitors in 2017 and a comparison with peptidases in the PANTHER database
Source: Nucleic Acids Res. 2017 Nov 14;46(Database issue):D624–32. doi: 10.1093/nar/gkx1134 (PMC5753285; doi:10.1093/nar/gkx1134)
Supplement: Supplementary Data [file gkx1134_supp.docx]

**Supplementary Table 1. A comparison of peptidase clans and families from archaeota, a bacterium and a eukaryote.**

Species shown are four from the Asgard superphylum of archaeota (Odinarchaeota archaeon LCB 4, Heimdallarchaeota archaeon AB 125, Thorarchaeota archaeon AB 25, Lokiarchaeota archaeon CR 4); the euryarchaeote *Pyrococcus furiosus*; the crenarchaeote *Sulfolobus acidocaldarius*; the pelagic alpha-proteobacterium *Pelagibacter ubique*; and the fission yeast *Schizosaccharomyces pombe.* Numbers of homologues are shown for each peptidase family and include sequences known or predicted to be peptidases (*i.e.* containing the full complement of active site residues) and non-peptidase homologues (*i.e.* where one of more active site residues is missing or has been replaced). A family restricted to one organism is highlighted in brown. A family with homologues from all species is highlighted in yellow, a family with homologues from Asgard archaeota and *S. pombe* only is highlighted in blue, a family with homologues from *Pelagibacter ubique* and *S. pombe* only is highlighted in orange. The total number of peptidase homologues and the total number of protein coding genes per proteome is also shown, along with the percentage of genes that encode peptidase homologues.

| **Clan** | **Family** | **Odinarchaeota** | **Heimdallarchaeota** | **Thorarchaeota** | **Lokiarchaeota** | ***P. furiosus*** | ***S. acidocaldarius*** | ***P. ubique*** | ***S. pombe*** |
| --- | --- | --- | --- | --- | --- | --- | --- | --- | --- |
| A- | A5 |  |  |  |  |  | 10 |  |  |
| A- | A37 |  |  |  |  |  | 7 |  |  |
| AA | A1 |  |  |  |  |  |  |  | 2 |
| AA | A2 |  |  |  |  |  |  |  | 6 |
| AA | A28 |  |  |  |  |  |  |  | 2 |
| [AC](http://ves-hx-b3.ebi.ac.uk/merops/cgi-bin/clansum?clan=AC) | [A8](http://ves-hx-b3.ebi.ac.uk/merops/cgi-bin/famsum?family=A8) |  |  |  |  |  |  | 2 |  |
| AD | A22 | 1 |  |  | 1 |  |  |  | 1 |
| AD | A24 |  |  |  |  |  | 2 | 2 |  |
| [AE](http://ves-hx-b3.ebi.ac.uk/merops/cgi-bin/clansum?clan=AE) | [A31](http://ves-hx-b3.ebi.ac.uk/merops/cgi-bin/famsum?family=A31) | 1 | 1 | 1 | 1 | 2 |  |  |  |
| CA | C12 |  |  |  |  |  |  |  | 2 |
| CA | C19 |  |  |  |  |  |  |  | 16 |
| CA | C54 |  |  |  |  |  |  |  | 1 |
| CA | C83 |  |  |  |  |  |  |  | 1 |
| CA | C85 |  |  |  |  |  |  |  | 2 |
| CA | C110 |  |  |  |  |  |  |  | 1 |
| CA | C115 |  |  |  |  |  |  |  | 1 |
| [CD](http://ves-hx-b3.ebi.ac.uk/merops/cgi-bin/clansum?clan=CD) | [C11](http://ves-hx-b3.ebi.ac.uk/merops/cgi-bin/famsum?family=C11) |  | 1 |  |  |  |  |  |  |
| CD | C13 |  |  |  |  |  |  |  | 1 |
| CD | C14 |  | 1 |  | 1 |  |  |  | 1 |
| CD | C25 |  |  | 2 | 1 |  |  |  |  |
| CD | C50 |  |  |  |  |  |  |  | 1 |
| CE | C48 |  |  |  |  |  |  |  | 4 |
| CF | C15 |  |  |  |  | 1 | 2 |  |  |
| CO | C40 |  |  |  |  |  |  | 2 |  |
| CP | C97 |  |  |  |  |  |  |  | 1 |
| [M-](http://ves-hx-b3.ebi.ac.uk/merops/cgi-bin/clansum?clan=M-) | [M79](http://ves-hx-b3.ebi.ac.uk/merops/cgi-bin/famsum?family=M79) | 1 |  | 3 | 1 |  | 1 |  | 1 |
| M- | M82 |  |  |  |  | 1 |  | 2 |  |
| [MA](http://ves-hx-b3.ebi.ac.uk/merops/cgi-bin/clansum?clan=MA) | [M1](http://ves-hx-b3.ebi.ac.uk/merops/cgi-bin/famsum?family=M1) | 1 | 1 |  |  | 3 | 1 |  | 3 |
| [MA](http://ves-hx-b3.ebi.ac.uk/merops/cgi-bin/clansum?clan=MA) | [M3](http://ves-hx-b3.ebi.ac.uk/merops/cgi-bin/famsum?family=M3) |  | 1 | 2 |  |  |  |  | 1 |
| MA | M12 |  |  |  |  |  |  |  | 1 |
| MA | M30 |  | 1 |  |  |  |  |  |  |
| MA | M32 |  | 1 | 1 | 1 | 1 | 1 |  |  |
| MA | M41 |  |  |  |  |  |  | 2 | 2 |
| MA | M48 | 1 | 1 | 1 |  | 2 | 4 | 1 | 2 |
| [MA](http://ves-hx-b3.ebi.ac.uk/merops/cgi-bin/clansum?clan=MA) | [M54](http://ves-hx-b3.ebi.ac.uk/merops/cgi-bin/famsum?family=M54) | 1 |  |  | 1 | 2 | 1 |  |  |
| MA | M61 |  |  |  |  |  | 1 | 1 |  |
| MA | M76 |  |  |  |  |  |  |  | 1 |
| MA | M80 |  |  |  |  |  |  |  | 2 |
| [MC](http://ves-hx-b3.ebi.ac.uk/merops/cgi-bin/clansum?clan=MC) | [M14](http://ves-hx-b3.ebi.ac.uk/merops/cgi-bin/famsum?family=M14) |  | 2 | 1 |  |  |  |  | 1 |
| ME | M16 |  |  |  |  |  |  | 2 | 8 |
| MF | M17 |  |  |  |  |  |  | 1 | 1 |
| [MG](http://ves-hx-b3.ebi.ac.uk/merops/cgi-bin/clansum?clan=MG) | [M24](http://ves-hx-b3.ebi.ac.uk/merops/cgi-bin/famsum?family=M24) | 2 | 5 | 7 | 4 | 5 | 3 | 5 | 8 |
| MH | M18 |  |  |  |  |  |  |  | 1 |
| [MH](http://ves-hx-b3.ebi.ac.uk/merops/cgi-bin/clansum?clan=MH) | [M20](http://ves-hx-b3.ebi.ac.uk/merops/cgi-bin/famsum?family=M20) | 1 | 7 | 5 | 4 | 5 | 8 | 5 | 3 |
| [MH](http://ves-hx-b3.ebi.ac.uk/merops/cgi-bin/clansum?clan=MH) | [M28](http://ves-hx-b3.ebi.ac.uk/merops/cgi-bin/famsum?family=M28) | 1 | 3 | 6 | 1 | 1 |  |  | 5 |
| [MH](http://ves-hx-b3.ebi.ac.uk/merops/cgi-bin/clansum?clan=MH) | [M42](http://ves-hx-b3.ebi.ac.uk/merops/cgi-bin/famsum?family=M42) | 1 | 3 | 1 | 2 | 4 |  |  |  |
| MJ | M19 |  |  |  |  | 1 | 1 | 2 | 2 |
| [MJ](http://ves-hx-b3.ebi.ac.uk/merops/cgi-bin/clansum?clan=MJ) | [M38](http://ves-hx-b3.ebi.ac.uk/merops/cgi-bin/famsum?family=M38) | 2 | 8 | 8 | 1 | 2 | 7 |  | 4 |
| [MM](http://ves-hx-b3.ebi.ac.uk/merops/cgi-bin/clansum?clan=MM) | [M50](http://ves-hx-b3.ebi.ac.uk/merops/cgi-bin/famsum?family=M50) | 2 | 1 | 2 | 2 | 3 | 2 | 3 |  |
| [MN](http://ves-hx-b3.ebi.ac.uk/merops/cgi-bin/clansum?clan=MN) | [M55](http://ves-hx-b3.ebi.ac.uk/merops/cgi-bin/famsum?family=M55) |  | 1 |  |  | 1 |  |  |  |
| [MO](http://ves-hx-b3.ebi.ac.uk/merops/cgi-bin/clansum?clan=MO) | [M23](http://ves-hx-b3.ebi.ac.uk/merops/cgi-bin/famsum?family=M23) |  | 3 | 5 | 5 |  |  | 2 |  |
| [MP](http://ves-hx-b3.ebi.ac.uk/merops/cgi-bin/clansum?clan=MP) | [M67](http://ves-hx-b3.ebi.ac.uk/merops/cgi-bin/famsum?family=M67) | 1 | 2 |  | 8 | 2 |  |  | 6 |
| MQ | M29 |  |  | 2 |  |  |  |  |  |
| [PA](http://ves-hx-b3.ebi.ac.uk/merops/cgi-bin/clansum?clan=PA) | [S1](http://ves-hx-b3.ebi.ac.uk/merops/cgi-bin/famsum?family=S1) |  | 1 | 2 |  | 1 | 1 | 3 | 3 |
| [PB](http://ves-hx-b3.ebi.ac.uk/merops/cgi-bin/clansum?clan=PB) | [C44](http://ves-hx-b3.ebi.ac.uk/merops/cgi-bin/famsum?family=C44) | 3 | 1 | 2 | 4 | 5 | 5 | 5 | 3 |
| PB | S45 |  |  | 1 | 1 |  | 1 |  |  |
| [PB](http://ves-hx-b3.ebi.ac.uk/merops/cgi-bin/clansum?clan=PB) | [T1](http://ves-hx-b3.ebi.ac.uk/merops/cgi-bin/famsum?family=T1) | 5 | 2 | 4 | 4 | 3 | 3 | 2 | 14 |
| [PB](http://ves-hx-b3.ebi.ac.uk/merops/cgi-bin/clansum?clan=PB) | [T2](http://ves-hx-b3.ebi.ac.uk/merops/cgi-bin/famsum?family=T2) |  |  | 2 |  | 1 | 1 |  | 1 |
| PB | T3 |  | 1 |  |  |  | 1 |  | 2 |
| [PC](http://ves-hx-b3.ebi.ac.uk/merops/cgi-bin/clansum?clan=PC) | [C26](http://ves-hx-b3.ebi.ac.uk/merops/cgi-bin/famsum?family=C26) | 4 |  | 1 | 3 | 7 | 4 | 6 | 8 |
| PC | C56 | 1 |  | 1 |  | 1 | 1 |  | 6 |
| PD | N10 | 1 |  |  | 1 | 8 |  |  |  |
| PE | P1 |  | 1 |  |  | 1 | 1 | 2 |  |
| PE | T5 |  |  |  | 1 |  |  |  | 1 |
| [SB](http://ves-hx-b3.ebi.ac.uk/merops/cgi-bin/clansum?clan=SB) | [S8](http://ves-hx-b3.ebi.ac.uk/merops/cgi-bin/famsum?family=S8) | 1 | 4 | 6 | 6 | 3 | 1 |  | 4 |
| SB | S53 | 1 |  |  |  |  | 3 |  |  |
| [SC](http://ves-hx-b3.ebi.ac.uk/merops/cgi-bin/clansum?clan=SC) | [S9](http://ves-hx-b3.ebi.ac.uk/merops/cgi-bin/famsum?family=S9) | 1 | 4 | 8 | 9 | 3 | 6 | 4 | 10 |
| SC | S10 |  |  |  |  |  |  |  | 3 |
| SC | S15 |  | 1 |  | 1 | 1 |  | 3 |  |
| [SC](http://ves-hx-b3.ebi.ac.uk/merops/cgi-bin/clansum?clan=SC) | [S33](http://ves-hx-b3.ebi.ac.uk/merops/cgi-bin/famsum?family=S33) | 1 | 10 | 11 | 12 | 1 | 8 | 5 | 10 |
| SE | S11 |  |  |  | 1 |  |  | 2 |  |
| [SE](http://ves-hx-b3.ebi.ac.uk/merops/cgi-bin/clansum?clan=SE) | [S12](http://ves-hx-b3.ebi.ac.uk/merops/cgi-bin/famsum?family=S12) |  | 5 | 8 | 6 |  |  |  |  |
| SF | S24 |  |  |  |  |  |  | 4 |  |
| [SF](http://ves-hx-b3.ebi.ac.uk/merops/cgi-bin/clansum?clan=SF) | [S26](http://ves-hx-b3.ebi.ac.uk/merops/cgi-bin/famsum?family=S26) | 1 |  | 2 | 3 | 2 | 2 | 2 | 3 |
| SJ | S16 | 1 | 1 |  |  | 2 |  | 2 | 1 |
| SK | S14 |  |  |  |  |  |  | 2 |  |
| SK | S41 |  |  |  |  |  | 1 | 3 |  |
| SK | S49 |  |  | 1 |  | 3 |  | 2 |  |
| SP | S59 |  |  |  |  |  |  |  | 1 |
| [SS](http://ves-hx-b3.ebi.ac.uk/merops/cgi-bin/clansum?clan=SS) | [S66](http://ves-hx-b3.ebi.ac.uk/merops/cgi-bin/famsum?family=S66) |  | 2 |  |  |  |  |  |  |
| ST | S54 | 1 | 1 |  | 1 | 1 | 1 |  | 4 |
| U- | U32 |  |  |  | 2 |  |  |  |  |
| [U-](http://ves-hx-b3.ebi.ac.uk/merops/cgi-bin/clansum?clan=U-) | [U62](http://ves-hx-b3.ebi.ac.uk/merops/cgi-bin/famsum?family=U62) | 4 | 7 | 9 | 4 | 6 | 5 | 2 |  |
| U- | U71 |  |  | 1 | 2 |  |  |  |  |
| Total |  | 41 | 84 | 105 | 93 | 85 | 79 | 81 | 159 |
| Proteins |  | 1584 | 2348 | 2914 | 4413 | 2121 | 2243 | 1393 | 5133 |
| % |  | 2.59 | 3.58 | 3.6 | 2.11 | 4.01 | 3.52 | 2.59 | 3.1 |
